# Characterization of the complete mitochondrial genome of *Spirocerca lupi*: sequence, gene organization and phylogenetic implications

**DOI:** 10.1186/1756-3305-6-45

**Published:** 2013-02-22

**Authors:** Guo-Hua Liu, Yan Wang, Hui-Qun Song, Ming-Wei Li, Lin Ai, Xing-Long Yu, Xing-Quan Zhu

**Affiliations:** 1 College of Veterinary Medicine, Hunan Agricultural University, Changsha, Hunan Province, 410128, China; 2 State Key Laboratory of Veterinary Etiological Biology, Key Laboratory of Veterinary Parasitology of Gansu Province, Lanzhou Veterinary Research Institute, Chinese Academy of Agricultural Sciences, Lanzhou, Gansu Province, 730046, China; 3 College of Veterinary Medicine, South China Agricultural University, Guangzhou, Guangdong Province, 510642, China; 4 Department of Veterinary Medicine, Agricultural College, Guangdong Ocean University, Huguangyan, Zhanjiang, Guangdong Province, 524088, China; 5 National Institute of Parasitic Diseases, Chinese Center for Disease Control and Prevention, WHO Collaborating Center for Malaria, Schistosomiasis and Filariasis, Key Laboratory of Parasite and Vector Biology, Ministry of Health, Shanghai, 200025, China

**Keywords:** *Spirocerca lupi*, Spirocercosis, Mitochondrial genome, Gene organization, Phylogenetic implication

## Abstract

**Background:**

*Spirocerca lupi* is a life-threating parasitic nematode of dogs that has a cosmopolitan distribution but is most prevalent in tropical and subtropical countries. Despite its veterinary importance in canids, the epidemiology, molecular ecology and population genetics of this parasite still remain unexplored.

**Methods:**

The complete mitochondrial (mt) genome of *S*. *lupi* was amplified in four overlapping long fragments using primers designed based on partial *cox*1, *rrn*S, *cox*2 and *nad*2 sequences. Phylogenetic re-construction of 13 spirurid species (including *S*. *lupi*) was carried out using Bayesian inference (BI) based on concatenated amino acid sequence datasets.

**Results:**

The complete mt genome sequence of *S*. *lupi* is 13,780 bp in length, including 12 protein-coding genes, 22 transfer RNA genes and two ribosomal RNA genes, but lacks the *atp*8 gene. The gene arrangement is identical to that of *Thelazia callipaeda* (Thelaziidae) and *Setaria digitata* (Onchocercidae), but distinct from that of *Dracunculus medinensis* (Dracunculidae) and *Heliconema longissimum* (Physalopteridae). All genes are transcribed in the same direction and have a nucleotide composition high in A and T. The content of A + T is 73.73% for *S*. *lupi*, in accordance with mt genomes of other spirurid nematodes sequenced to date. Phylogenetic analyses using concatenated amino acid sequences of the 12 protein-coding genes by BI showed that the *S*. *lupi* (Thelaziidae) is closely related to the families Setariidae and Onchocercidae.

**Conclusions:**

The present study determined the complete mt genome sequence of *S*. *lupi*. These new mt genome dataset should provide novel mtDNA markers for studying the molecular epidemiology and population genetics of this parasite, and should have implications for the molecular diagnosis, prevention and control of spirocercosis in dogs and other canids.

## Background

The nematode *Spirocerca lupi* (Rudolphi, 1809) (at the adult stage) parasitizes the oesophagus and aorta of canids, especially in dogs. *S*. *lupi* is responsible for canine spirocercosis with a worldwide distribution but is usually found in tropical and subtropical countries [1,2]. Canine spirocercosis is usually associated with several clinical signs, such as regurgitation, vomiting and dyspnoea [3,4]. This disease is also fatal when it causes malignant neoplasms or aortic aneurysms [2,4,5]. Fortunately, spirocercosis can be treated efficiently using anthelminthics, such as doramectin [6].

Canine spirocercosis caused by *S*. *lupi* is often neglected and underestimated by some veterinary scientists and practitioners. However, *S*. *lupi* is most prevalent in dogs in rural areas, such as in Bangladesh (40%) [7], Greece (10%) [8], Grenada (8.8% in owned dogs and 14.2% in stray dogs) [1], India (23.5%) [9], Iran (19%) [10], South Africa (13%) [11] and Kenya (85% in stray dogs and 38% in owned dogs) [12]. *S*. *lupi* has been also reported in dogs in China, with a very high prevalence (78.6%) [13]. Although canine spirocercosis is an emerging disease, little is known about the molecular biology and genetics of *S*. *lupi*[14]. A previous study has found utility of mitochondrial (mt) cytochrome *c* oxidase subunit 1 (*cox*1) for population genetic and phylogenetic studies of *S*. *lupi*[14], yet, there is still a paucity of information on *S*. *lupi* mt genomics.

mt genome sequences provide useful genetic markers not only for genetic and epidemiological investigations and molecular identification of parasites, but also for phylogenetic and population studies [15-18] due to its maternal inheritance, rapid evolutionary rate, and lack of recombination [19,20]. To date, although mt genome sequences have been sequenced for 12 species within the order Spirurida, only one mt genome (for *Thelazia callipaeda*) is available within the family Thelaziidae [21]. Therefore, the objectives of the present study were to determine the complete mt genome sequence of *S*. *lupi* and to assess the phylogenetic position of this nematode in relation to other spirurid nematodes for which complete mt sequence datasets are available.

## Methods

### Ethics statement

This study was approved by the Animal Ethics Committee of Lanzhou Veterinary Research Institute, Chinese Academy of Agricultural Sciences (Approval No. LVRIAEC2010-007). The farmed dog from which *S*. *lupi* adults were collected, was handled in accordance with good animal practices required by the Animal Ethics Procedures and Guidelines of the People’s Republic of China.

### Parasites and DNA extraction

Adult nematodes representing *S*. *lupi* were obtained at *post mortem* from the oesophagus of an infected farmed dog in Zhanjiang, Guangdong province, China. These specimens were washed in physiological saline, identified morphologically to species according to existing descriptions [22], fixed in 70% (v/v) ethanol and stored at −20°C until use.

Total genomic DNA was isolated from one *S*. *lupi* worm using sodium dodecyl sulphate/proteinase K treatment, followed by spin-column purification (TIANamp Genomic DNA kit). In order to independently verify the identity of this specimen, the mt *cox*1 gene was amplified by the polymerase chain reaction (PCR) and sequenced according to an established method [14]. The *cox*1 sequence of this *S*. *lupi* sample had 96.5% similarity with that of *S*. *lupi* in dogs in South Africa (GenBank accession no. HQ674759).

### Amplification and sequencing of partial *cox*1, *rrn*S, *cox*2 and *nad*2 genes

Initially, a fragment of *cox*1 (346 bp) was amplified by conserved primers JB3/JB4.5 [23], and *rrn*S (213 bp), *cox*2 (300 bp) and *nad*2 (1200 bp) were amplified by PCR with primers designed (Table 1) based on sequences well conserved in many related taxa. PCR reactions (25 mL) were performed in 10 mM Tris–HCl (pH 8.4), 50 mM KCl, 4 mM MgCl2, 200 mM each of dNTP, 50 pmol of each primer and 2 U *Taq* polymerase (Takara) in a thermocycler (Biometra) under the following conditions: after an initial denaturation at 94°C for 5 min, then 94°C for 30 s (denaturation), 55°C (for *cox*1) or 48°C (for *cox*2) or 50°C (for *nad*2 and *rrn*S) for 30 s (annealing), 72°C for 1 min (extension) for 36 cycles, followed by 72°C for 10 min (final extension). Two microliters (5–10 ng) of genomic DNA was added to each PCR reaction. Each amplicon (5 μL) was examined by agarose gel electrophoresis to validate amplification efficiency. Then, these amplicons were sent to Sangon Company (Shanghai, China) for sequencing from both directions by using primers used in PCR amplifications.

**Table 1:** **Sequences of primers used to amplify PCR fragments from *Spirocerca lupi***

**Name of primer**	**Sequence (5’ to 3’)**
Short-PCR	
For *cox*1	
JB3	TTTTTTGGGCATCCTGAGGTTTAT
JB4.5	TAAAGAAAGAACATAATGAAAATG
For *cox*2	
SLCO2F	TTGAAATTACGAGTATGGGGATA
SLCO2R	AGCTCCACAAATTTCTGAACACT
For *nad*2	
SLND2F	TGGTGGAGGGGTTTTGTTATTTG
SLND2R	ATCTTCTCAACCTGACGACC
For *rrn*S	
SL12SF	AATCAAAATTTATTAGTTCGGGAGT
SL12SR	AATTACTTTTTTTTCCAACTTCAA
Long-PCR	
SLCO1F	CTTTAGGTGGTTTGAGAGGTATTGTT
SL12S R	CTTCATAAACCAAATATCTATCTGT
SL12SF	ATAGATATTTGGTTTATGAAGATTT
SLCO2R	AAGAATGAATAACATCCGAAGAAGT
SLCO2F	CCTATTGTTGGCTTATTTTATGGTCAG
SLND2R	CAAAAATGAAAAGGTGCCGAACCAGAT
SLND2F	GGTTTTGGTCGTCAGGTTGAGAAGA
SLCO1R	ATCATAGTAGCCGCCCTAAAATAAGTA

### Long-PCR amplification and sequencing

After we had obtained partial *cox*1, *rrn*S, *cox*2 and *nad*2 sequences for the *S*. *lupi*, we then designed four primers (Table 1) in the conserved regions to amplify the entire mt genome of *S*. *lupi* from this representative sample in four overlapping long fragments between *cox*1 and *rrn*S (approximately 4.5 kb), between *rrn*S and *cox*2 (approximately 2.5 kb), between *cox*2 and *nad*2 (approximately 4 kb), and between *nad*2 and *cox*1 (approximately 3 kb). Long-PCR reactions (25 μl) were performed in 2 mM MgCl_2_, 0.2 mM each of dNTPs, 2.5 μl 10× LA *Taq* buffer, 2.5 μM of each primer, 1.25 U LA *Taq* polymerase (Takara), and 2 μl of DNA sample in a thermocycler (Biometra) under the following conditions: 92°C for 2 min (initial denaturation), then 92°C for 10 s (denaturation), 60°C (for 4.5 kb) or 44°C (for 2.5 kb) or 52°C (for 4 kb) or 48°C (for 3 kb fragment) for 30 s (annealing), and 60°C for 10 min (extension) for 10 cycles, followed by 92°C for 10 s, 60°C (for 4.5 kb) or 44°C (for 2.5 kb) or 52°C (for 4 kb) or 48°C (for 3 kb fragment) for 30 s (annealing), and 60°C for 10 min for 20 cycles, with a cycle elongation of 10 s for each cycle and a final extension at 60°C for 10 min. Each PCR reaction yielded a single band detected in a 0.8% (w/v) agarose gel (not shown). PCR products were sent to Sangon Company (Shanghai, China) for sequencing using a primer-walking strategy.

### Sequence analyses

Sequences were assembled manually using the commercial software ContigExpress program of the Vector NTI software package version 6.0 (Invitrogen, Carlsbad, CA), and aligned against the complete mt genome sequences of other spirurid nematodes available using the computer program Clustal X 1.83 [24] and MegAlign procedure within the DNAStar 5.0 [25] to infer gene boundaries. The open-reading frames were analysed with Open Reading Frame Finder (http://www.ncbi.nlm.nih.gov/gorf/gorf.html) using the invertebrate mitochondrial code, and subsequently compared with that of *T*. *callipaeda*[21]. Protein-coding gene sequences were translated into amino acid sequences using the invertebrate mitochondrial genetic code; amino acid sequences were aligned using default settings with MEGA 5.0 [26]. Translation initiation and termination codons were identified by comparison with those of the spirurid nematodes reported previously [21,27]. For analyzing ribosomal RNA genes, putative secondary structures of 22 tRNA genes were identified using tRNAscan-SE [28], of the 22 tRNA genes, 14 were identified using tRNAscan-SE, the other 8 tRNA genes were found by eye inspection, and rRNA genes were identified by comparison with that of spirurid nematodes [21,27].

### Phylogenetic analysis

The amino acid sequences conceptually translated from individual genes of the mt genome of *S*. *lupi* were concatenated. Selected for comparison were concatenated amino acid sequences predicted from published mt genomes of key nematodes representing the order Spirurida, including the superfamilies Thelazoidea (*T*. *callipaeda*[21]), Filarioidea (*Acanthocheilonema viteae*[29], *Brugia malayi*[30], *Chandlerella quiscali*[29], *Dirofilaria immitis*[31], *Loa loa*[29], *Onchocerca flexuosa*[29], *O*. *volvulus*[32], *S*. *digitata*[27] and *Wuchereria bancrofti*[18]), Dracunculoidea (*Dracunculus medinensis*[33]) and Physalopteroidea (*Heliconema longissimum*[33]) (GenBank accession numbers JX069968, NC_016197, NC_004298, NC_014486, NC_005305, NC_016199, NC_016172, AF015193, NC_014282, JN367461, NC_016019 and NC_016127, respectively), using *Ascaris suum*[34] (GenBank accession number HQ704901) as the outgroup. The amino acid sequences were aligned using Clustal X 1.83 [24] using default settings, ambiguously aligned regions were excluded using Gblocks online server (http://molevol.cmima.csic.es/castresana/Gblocks_server.html) using the options for a less stringent selection, and then subjected to phylogenetic analysis using Bayesian inference (BI) as described previously [35,36]. Phylograms were drawn using the Tree View program v.1.65 [37].

## Results and discussion

### General features of the *S*. *lupi* mt genome

**Figure 1:**
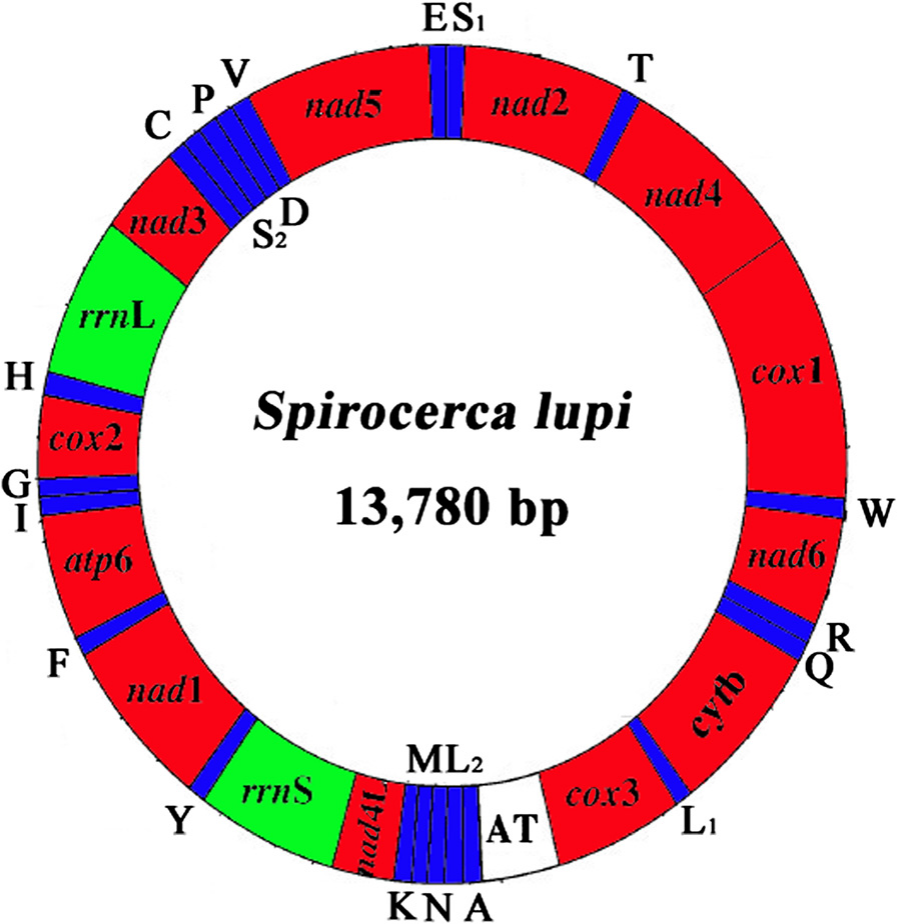
**Arrangement of the mitochondrial genome of *Spirocerca lupi. ***Gene scaling is only approximate. All genes are coded by the same DNA strand and are transcribed clockwise. All genes have standard nomenclature except for the 22 tRNA genes, which are designated by the one-letter code for the corresponding amino acid, with numerals differentiating each of the two leucine- and serine-specifying tRNAs (L_1_ and L_2_ for codon families CUN and UUR, respectively; S_1_ and S_2_ for codon families UCN and, AGN respectively). “AT” refers to the non-coding region.

The complete mtDNA sequence of *S*. *lupi* was 13,780 bp in size (Figure 1), and has been deposited in the GenBank under the accession number KC305876. The mt genome of *S*. *lupi* contains 12 protein-coding genes (*cox*1-3, *nad*1-6, *nad*4L, *atp*6 and *cyt*b), 22 transfer RNA genes, two ribosomal RNA genes (*rrn*L and *rrn*S) and a non-coding (control or AT-rich) region, but lacks an *atp*8 gene (Table 2). All genes are transcribed in the same direction. The gene order is identical to those of *T*. *callipaeda* and *S*. *digitata*[21,27], but distinct from those of *H*. *longissimum* (rearrangement markedly) and *Dracunculus medinensis* (tRNA-Met and tRNA-Val change) [33]. The nucleotide compositions of *S*. *lupi* mt genome is biased toward A and T, with T being the most favored nucleotide and C being the least favored, in accordance with mt genomes of other spirurid nematodes [27,31]. The content of A + T is 73.73% for *S*. *lupi*, similar to that of mt genomes of other spirurid nematodes sequenced to date, such as that of *T*. *callipaeda* (74.57%) [21] and *W*. *bancrofti* (74.59%) [18] (Table 3). Furthermore, the *S*. *lupi* mt genes overlap a total of 98 bp in 16 locations ranging from 1 to 32 bp (Table 2). The longest is a 32 bp overlap between *nad*1 and tRNA-Phe. The mt genome of *S*. *lupi* has 150 bp of intergenic regions at 16 locations ranging in size from 1 bp to 59 bp, the longest intergenic region is a 59 bp between tRNA-Pro and tRNA-Asp (Table 2). The mt genome of *T*. *callipaeda* has 14 intergenic regions, which range from 1 to 62 bp in length. The longest region is 62 bp between tRNA-Pro and tRNA-Asp [21].

**Table 2:** **Mitochondrial genome organization of *Spirocerca lupi***

**Gene/region**	**Positions**	**Size (bp)**	**Number of aa**^**a**^	**Ini/Ter codons**	**Anticodons**	**In**
*cox*1	1-1650	1650	549	ATG/TAA		+7
tRNA-Trp (W)	1657-1714	58			TCA	+6
*nad*6	1751-2209	459	152	TTG/TAA		+36
tRNA-Arg (R)	2207-2266	60			ACG	−3
tRNA-Gln (Q)	2263-2316	54			TTG	−4
*cyt*b	2315-3397	1083	360	ATT/TAA		−2
tRNA-LeuCUN (L_1_)	3396-3450	55			TAG	−2
*cox*3	3448-4230	783	260	ATA/TAA		−3
Non-coding region	4231-4630	400				0
tRNA-Ala (A)	4631-4692	62			TGC	0
tRNA-LeuUUR (L_2_)	4689-4742	54			TAA	−4
tRNA-Asn (N)	4747-4804	58			GTT	+4
tRNA-Met (M)	4807-4864	58			CAT	+2
tRNA-Lys (K)	4867-4924	58			TTT	+2
*nad*4L	4932-5159	228	75	ATG/TAG		+7
*rrn*S	5170-5855	686				+10
tRNA-Tyr (Y)	5855-5910	56			GTA	−1
*nad*1	5908-6816	909	302	TTG/TAA		−3
tRNA-Phe (F)	6785-6843	59			TTG	−32
*atp*6	6847-7431	585	194	ATT/TAG		+3
tRNA-Ile (I)	7435-7491	57			GAT	+3
tRNA-Gly (G)	7492-7546	55			TCC	0
*cox*2	7549-8253	705	234	ATG/TAG		+2
tRNA-His (H)	8244-8302	59			GTG	−10
*rrn*L	8301-9288	988				−2
*nad*3	9281-9616	336	111	TTG/TAA		−8
tRNA-Cys (C)	9616-9670	55			GCA	−1
tRNA-SerUCN (S_2_)	9673-9726	54			TGA	+2
tRNA-Pro (P)	9730-9787	58			AGG	+3
tRNA-Asp (D)	9847-9900	54			GTC	+59
tRNA-Val (V)	9902-9955	54			TAC	+1
*nad*5	9959-11551	1593	530	TTG/TAG		+3
tRNA-Glu (E)	11550-11606	57			TTC	−2
tRNA-SerAGN (S_1_)	11607-11656	50			TCT	0
*nad*2	11637-12485	849	282	ATG/TAG		−20
tRNA-Thr (T)	12487-12543	57			TGT	−1
*nad*4	12544-13773	1230	409	TTG/TAG		0

^a^The inferred length of amino acid sequence of 12 protein-coding genes; Ini/Ter codons: initiation and termination codons; In: Intergenic nucleotides.

**Table 3:** **Comparison of A + T content (%) of gene and region of the mt genomes of spirurid nematodes sequenced to date (alphabetical order), including *Spirocerca lupi *(in bold)**

**Gene/region**	**AV**	**BM**	**CQ**	**DI**	**DM**	**HL**	**LL**	**OF**	**OV**	**SD**	**SL**	**TC**	**WB**
*atp*6	75.21	75.09	80.14	71.88	72.40	77.89	76.46	73.71	72.99	74.23	**74.87**	74.23	76.63
*cox*1	67.36	68.98	70.28	67.88	68.21	71.69	69.48	69.70	67.03	69.10	**66.97**	67.88	67.70
*cox*2	66.81	68.96	73.25	69.15	68.25	74.71	71.53	68.10	69.24	69.38	**68.51**	67.38	70.57
*cox*3	71.54	72.69	76.92	71.79	71.54	75.93	76.20	72.18	71.79	72.56	**71.39**	72.41	74.33
*cytb*	72.32	73.97	76.13	72.25	72.14	79.30	75.35	73.65	72.11	72.34	**72.85**	73.68	72.70
*nad*1	73.43	73.55	75.85	72.94	72.29	75.69	72.85	71.60	69.78	72.78	**72.50**	73.22	72.52
*nad*2	74.68	77.61	82.39	74.39	76.93	82.92	77.26	75.56	74.30	76.49	**70.91**	77.35	75.71
*nad*3	79.82	79.35	81.71	77.15	75.89	83.18	79.82	76.56	76.11	77.06	**80.65**	80.24	84.27
*nad*4	73.98	76.31	78.05	74.55	72.32	80.36	75.75	74.05	73.15	76.91	**74.47**	75.59	73.88
*nad*4L	76.89	82.08	83.33	77.37	74.39	82.05	81.09	77.73	78.60	76.76	**76.75**	80.17	80.66
*nad*5	71.93	74.81	78.17	73.75	73.64	78.93	74.03	73.62	72.87	74.81	**72.88**	73.82	74.69
*nad*6	77.19	81.46	82.89	80.57	76.26	81.74	81.98	81.11	79.11	82.44	**77.56**	80.17	80.04
*rrn*S	75.48	76.04	76.85	75.84	73.59	80.50	76.56	75.84	74.71	74.55	**76.09**	75.68	75.30
*rrn*L	77.78	80.78	80.25	79.55	76.70	81.81	78.65	77.71	76.95	79.40	**79.05**	77.43	79.01
AT-loop	83.37	85.11	86.49	85.91	74.75	96.75	83.68	79.93	85.32	86.36	**88.50**	79.57	83.71
Entire	73.54	75.46	77.67	74.16	72.72	79.11	75.54	74.17	73.30	75.14	**73.73**	74.57	74.59

*Nematodes: AV: *Acanthocheilonema viteae*, BM: *Brugia malayi*, CQ: *Chandlerella quiscali*, DI: *Dirofilaria immitis*, DM: *Dracunculus medinensis*, HL: *Heliconema longissimum*, LL: *Loa loa*, OF: *Onchocerca flexuosa*, OV: *Onchocerca volvulus*, SD: *Setaria digitata*, SL: *Spirocerca lupi*, TC: *Thelazia callipaeda*, WB: *Wuchereria bancrofti*, Entire: entire mt genome.

### Protein-coding genes

The *S*. *lupi* mt genome encodes 12 protein-coding genes, which are identical to those of *T*. *callipaeda* and *S*. *digitata*[21,27]. For *S*. *lupi*, the sizes of the protein-coding genes were in the order: *cox*1 > *nad*5 > *nad*4 > *cyt*b > *nad*1 > *nad*2 > *cox*3 > *cox*2 > *atp*6 > *nad*6 > *nad*3 > *nad*4L (Table 2). The predicted translation initiation and termination codons for the 12 protein-coding genes of *S*. *lupi* mt genome were compared with that of *T*. *callipaeda* and *S*. *digitata*[21,27]. The most common initiation codon for *S*. *lupi* is TTG (5 of 12 protein genes), followed by ATG (4 of 12 protein genes), ATT (2 of 12 protein genes) and ATA (1 of 12 protein genes) (Table 2). In this mt genome, all protein genes were predicted to have a TAA or TAG as termination codon (Table 2). Although incomplete termination codons (T or TA) are present in some other nematodes, including *Anisakis simplex* (*s*. *l*.) [38], *A*. *suum*[39], *Caenorhabditis elegans*[39], *S*. *digitata*[27], *Toxocara* spp. [40] and *Trichinella spiralis*[41], they were not identified in the *S*. *lupi* mt genome.

Excluding the termination codons, a total of 3,458 amino acids of protein-coding genes are encoded by the *S*. *lupi* mt genome. Table 4 shows the codon usage. Condons composed of A and T are predominantly used, which seems to reflect the high A + T content of the mt genome of *S*. *lupi*. A strong preference for A + T rich codons usage is found in mtDNA of *S*. *lupi*. For example, the most frequently used amino acid was Phe (TTT: 17.03%), followed by Leu (TTG: 6.77%), Tyr (ATA: 6.16%) and IIe (ATT: 6.10%). This result is consistent with a recent study [21].

**Table 4:** **Codon usage of *Spirocerca lupi *mitochondrial protein-coding genes**

**Amino acid**	**Codon**	**Number**	**Frequency (%)**	**Amino acid**	**Codon**	**Number**	**Frequency (%)**
Phe	TTT	591	17.03	Met	ATA	52	1.49
Phe	TTC	16	0.46	Met	ATG	103	2.96
Leu	TTA	195	5.61	Thr	ACT	81	2.33
Leu	TTG	235	6.77	Thr	ACC	3	0.08
Ser	TCT	139	4.00	Thr	ACA	2	0.05
Ser	TCC	7	0.20	Thr	ACG	3	0.08
Ser	TCA	8	0.23	Asn	AAT	87	2.50
Ser	TCG	6	0.17	Asn	AAC	6	0.17
Tyr	TAT	214	6.16	Lys	AAA	42	1.21
Tyr	TAC	6	0.17	Lys	AAG	56	1.61
Stop	TAA	7	0.20	Ser	AGT	99	2.85
Stop	TAG	5	0.14	Ser	AGC	5	0.14
Cys	TGT	75	2.16	Ser	AGA	22	0.63
Cys	TGC	3	0.08	Ser	AGG	30	0.86
Trp	TGA	36	1.03	Val	GTT	239	6.88
Trp	TGG	56	1.61	Val	GTC	5	0.14
Leu	CTT	19	0.54	Val	GTA	35	1.00
Leu	CTC	0	0	Val	GTG	37	1.06
Leu	CTA	10	0.28	Ala	GCT	64	1.84
Leu	CTG	2	0.05	Ala	GCC	4	0.11
Pro	CCT	55	1.58	Ala	GCA	1	0.02
Pro	CCC	7	0.20	Ala	GCG	10	0.28
Pro	CCA	6	0.17	Asp	GAT	66	1.90
Pro	CCG	9	0.25	Asp	GAC	2	0.05
His	CAT	52	1.49	Glu	GAA	31	0.89
His	CAC	1	0.02	Glu	GAG	42	1.21
Gln	CAA	20	0.57	Gly	GGT	143	4.12
Gln	CAG	31	0.89	Gly	GGC	12	0.34
Arg	CGT	46	1.32	Gly	GGA	33	0.95
Arg	CGC	1	0.02	Gly	GGG	72	2.07
Arg	CGA	3	0.08	IIe	ATT	212	6.10
Arg	CGG	6	0.17	IIe	ATC	4	0.11

Total number of codons is 3,470.

Stop = Stop codon.

### Transfer RNA genes and ribosomal RNA genes

The sizes of 22 tRNA genes identified in the *S*. *lupi* mt genome ranged from 50 to 62 bp in size. Secondary structures predicted for the 22 tRNA genes of *S*. *lupi* (not shown) are similar to that of *S*. *digitata*[27]. The *rrn*L and *rrn*S genes of *S*. *lupi* were identified by comparison with the mt genomes of *T*. *callipaeda* and *S*. *digitata*. The *rrn*L is located between tRNA-His and *nad*3, and *rrn*S is located between *nad*4L and tRNA-Tyr. The lengths of the *rrn*L and *rrn*S genes were 988 bp and 686 bp for *S*. *lupi*, respectively (Table 2). The A + T contents of the *rrn*L and *rrn*S genes for *S*. *lupi* are 79.05% and 76.09%, respectively.

### Non-coding regions

The majority of nematode mtDNA sequences contain usually two non-coding regions of significant size difference, the long non-coding region and the short non-coding region, including *A*. *lumbricoides* and *A*. *suum*[34], *Contracaecum rudolphii* B [42], *Oesophagostomum* spp. [43], *Toxocara* spp. [40] and *Trichuris* spp. [44,45]. However, there is only one non-coding region (AT-rich region) in the mt genome of *S*. *lupi*, which is located between *cox*3 and tRNA-Ala (Figure 1 and Table 2), with 88.50% A + T content (Table 3). This region of the mt genome of *S*. *lupi* was considered as a non-coding region (or AT-rich region) due to its location and AT rich feature based on comparison with those of spirurid nematodes reported previously [21,27]. Moreover, in the AT-rich region of *S*. *lupi* consecutive sequences [A]_13_ and [T]_12_ were found, but there are no AT dinucleotide repeat sequences similar to that of *A*. *simplex s*.*l*. and *S*. *digitata* in the this region [27,38].

### Phylogenetic analyses

**Figure 2:**
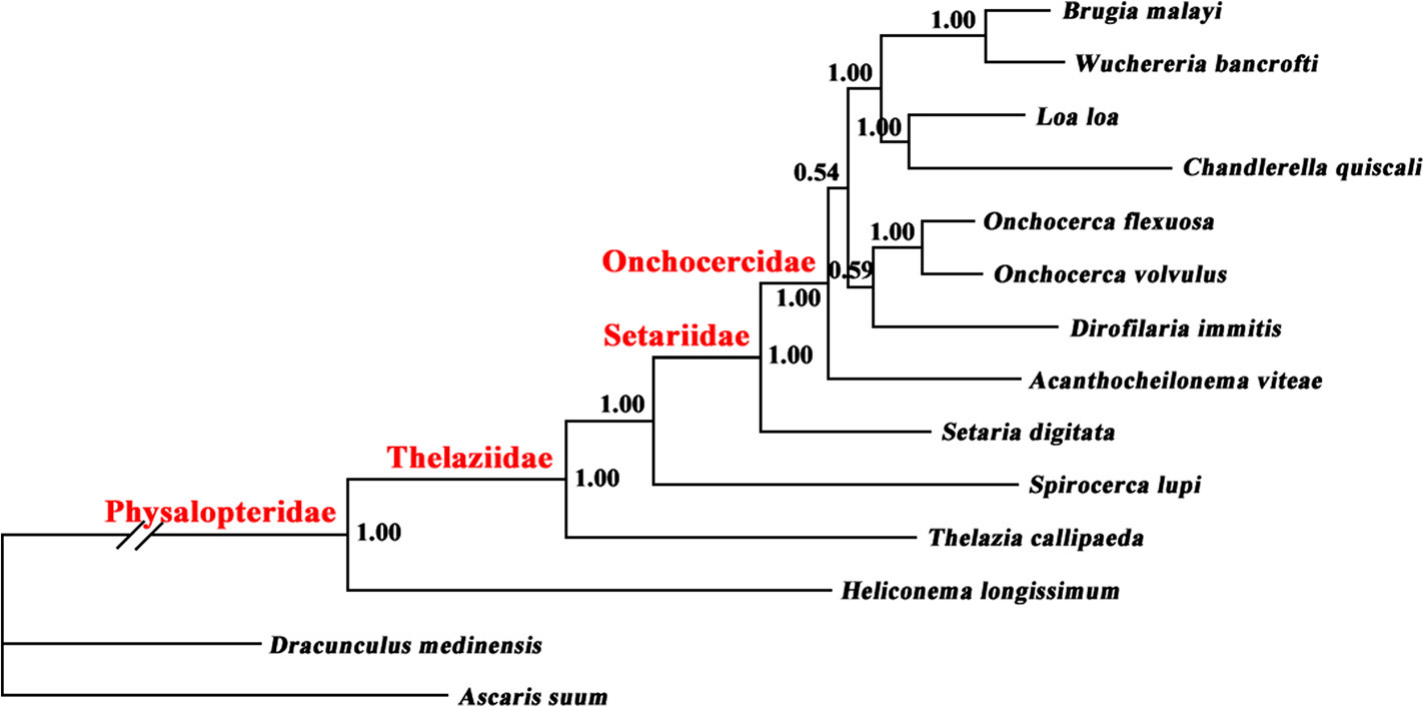
**Relationship of *Spirocerca lupi *with other selected spirurid nematodes based on mitochondrial sequence data.** The concatenated amino acid sequences of 12 protein-coding genes were subjected to analysis by Bayesian inference (BI) using *Ascaris suum* as the outgroup. Posterior probability (pp) values are indicated.

The phylogenetic relationships of 12 spirurid species based on concatenated amino acid sequence datasets, plus the mtDNA sequence of *S*. *lupi* obtained in the present study, using BI is shown in Figure 2. The results revealed that *S*. *lupi* (Thelaziidae) was a sister taxon to a clade containing *S*. *digitata* (Setariidae) and other members of the Onchocercidae, including *B*. *malayi* and *D*. *immitis* (posterior probability = 1.00), consistent with results of previous studies [14,21,46].

Many studies have demonstrated that mtDNA sequences are valuable genetic markers for phylogenetic studies of members within the Nematoda. A recent study analyzed mt sequence variations in human- and pig-derived *Trichuris* and demonstrated that they represent separate species [44]. In addition, a previous study sequenced and compared the mt genomes of *A*. *lumbricoides* and *A*. *suum* from humans and pigs and indicted that *A*. *lumbricoides* and *A*. *suum* may represent the same species [34]. In the present study, the characterization of the mt genome of *S*. *lupi* can promote to reassess the systematic relationships within the order Spirurida using mt genomic datasets. For many years, there have been considerable debates about the phylogenetic position of members of spirurid nematodes [47,48]. Given this utility of mt genomic datasets, thus, further work should sequence more mt genomes of spirurid nematodes and re-construct the phylogenetic relationships of spirurid nematodes using expanded mt datasets.

## Conclusions

The present study determined the complete mt genome sequence of *S*. *lupi*, and ascertained its phylogenetic position within the Spirurida. These new mtDNA data will provide useful novel markers for studying the molecular epidemiology and population genetics of *S*. *lupi*, and have implications for the diagnosis, prevention and control of spirocercosis in canid animals.

## Competing interests

The authors declare that they have no competing interests.

## Authors’ contributions

XQZ and XLY conceived and designed the study, and critically revised the manuscript. GHL, YW and HQS performed the experiments, analyzed the data and drafted the manuscript. MWL and LA helped in study design, study implementation and manuscript revision. All authors read and approved the final manuscript.
